# IMPLEMENTATION AND OUTCOMES OF DOLUTEGRAVIR-BASED FIRST-LINE ANTIRETROVIRAL THERAPY FOR PEOPLE WITH HIV IN SOUTH AFRICA: A RETROSPECTIVE COHORT STUDY

**DOI:** 10.1016/S2352-3018(23)00047-4

**Published:** 2023-03-28

**Authors:** Jienchi Dorward, Yukteshwar Sookrajh, Thokozani Khubone, Johan van der Molen, Riona Govender, Sifiso Phakathi, Lara Lewis, Christian Bottomley, Munthra Maraj, Richard J Lessells, Kogieleum Naidoo, Christopher C Butler, Rose van Heerden, Nigel Garrett

**Affiliations:** 1. Centre for the AIDS Programme of Research in South Africa (CAPRISA), University of KwaZulu–Natal, Durban, KwaZulu-Natal, South Africa; 2. Nuffield Department of Primary Care Health Sciences, University of Oxford, Oxford, Oxfordshire, United Kingdom; 3. eThekwini Municipality Health Unit, eThekwini Municipality, Durban KwaZulu-Natal, South Africa; 4. Health Informatics Directorate, South African National Department of Health, Pretoria, South Africa; 5. London School of Hygiene & Tropical Medicine, London, United Kingdom; 6. KwaZulu-Natal Research and Innovation Sequencing Platform (KRISP), University of KwaZulu-Natal, Durban, KwaZulu-Natal, South Africa; 7. South African Medical Research Council (SAMRC)-CAPRISA-TB-HIV Pathogenesis and Treatment Research Unit, University of KwaZulu-Natal Nelson R Mandela School of Medicine, Durban, South Africa; 8. Discipline of Public Health Medicine, School of Nursing and Public Health, University of KwaZulu-Natal, Durban, KwaZulu-Natal, South Africa

**Keywords:** HIV, dolutegravir, antiretroviral therapy, women, South Africa, retention-in-care, viral load

## Abstract

**Background:**

There is little data evaluating uptake of first-line dolutegravir among men and women living with HIV in low- and middle-income countries, and subsequent clinical outcomes in non-trial settings. We aimed to determine dolutegravir uptake in women, and the impact of dolutegravir on clinical outcomes in routine care in South Africa.

**Methods:**

We analysed de-identified data from adults receiving first-line ART at 59 South African clinics from December 2019 to February 2022, using two distinct cohorts. In the ‘initiator cohort’, we used Poisson regression models to assess initiation with dolutegravir-based ART by gender, and associations between dolutegravir use and 12-month retention-in-care and viral suppression <50 copies/mL. In the ‘transition cohort’, consisting of adults receiving non-dolutegravir-based first-line ART in December 2019, we used Cox proportional hazards models to assess the hazard of transition to first-line dolutegravir by gender. We then used time-dependent propensity score matching to compare subsequent 12-month retention-in-care and viral suppression between people transitioned to dolutegravir, and those who had not yet transitioned at the same timepoint.

**Findings:**

Of 45,392 adults initiating ART, 28,725 (63·3%) were women, median age was 31 years (IQR 26,38), and 31,264 (69·9%) initiated dolutegravir. Dolutegravir initiation was lower in non-pregnant (risk difference [RD] −18·4% [−21·6,−15·2]) and pregnant women (RD −35·4% [−42·3,−28·5]) versus men. At 12-months, retention-in-care (adjusted RD 5·2% [2·2,8·4)) and viral suppression (aRD 3·1% [1·2,5·1]) was higher in dolutegravir initiators.

Among 180,956 adults receiving first-line ART in December 2019, median age was 38 years (32,45), 124,168 (68·6%) were women and 98·6% received efavirenz. By February 2022, 121,210/158,999 (76·2%) of those in care were transitioned to first-line dolutegravir. Transition was lower in women (hazard ratio 0·56, [0·56,0·57]). Among 92,318 propensity score matched people, 12-month retention-in-care (aRD 2·5% [2·1–2·9]) and viral suppression (aRD 0.8% [0.3,1.4]) were slightly higher in the dolutegravir versus non-dolutegravir group.

**Interpretation:**

Women were less likely to receive dolutegravir. As dolutegravir was associated with better outcomes, the rollout should continue, with a particular emphasis on inclusion of women.

**Funding:**

Wellcome Trust; Africa Oxford Initiative; International Association of Providers of AIDS Care, Bill & Melinda Gates Foundation

## INTRODUCTION

Since 2018 the World Health Organization (WHO) has recommended dolutegravir-based first-line antiretroviral therapy (ART),^1^ due to clinical trial evidence of increased efficacy compared to non-nucleoside reverse transcriptase inhibitors (NNRTIs). This is largely driven by better tolerability and less discontinuations, and dolutegravir is also more robust against the development of HIV drug resistance.^2,3^ Initially, safety concerns regarding a potential increased risk of neural tube defects if dolutegravir was taken at conception, led the WHO to recommend restricted use among women of child-bearing potential.^1^ However, new data suggesting a lower risk of neural tube defects, risk-benefit analyses from modelling studies,^4^ and community advocacy^5^ led WHO to remove these restrictions in July 2019.^1^ By mid-2022 dolutegravir had been introduced into the preferred first-line ART regimen in approximately 108 countries.^6^

To date, there are few published studies examining the impact of the above restrictions on dolutegravir uptake over time,^7–9^ and little data comparing effectiveness of dolutegravir with alternative first-line regimens in non-trial settings in Africa.^10^ In regions with high prevalence of tuberculosis co-infection, there are also important concerns regarding the use of dolutegravir with rifampicin-containing tuberculosis treatment, which reduces dolutegravir drug levels. This can be overcome by doubling the dolutegravir dose,^11^ which may not always be feasible in large-scale ART programmes. Dolutegravir use is particularly pertinent in South Africa, where there are over 7·5 million PLHIV, the majority of whom are women of child-bearing potential, and tuberculosis incidence is high.

We aimed to assess the impact of the first-line dolutegravir rollout on HIV treatment outcomes in South Africa. We determined a) the extent to which women were less likely to receive dolutegravir over time, b) the relationship between dolutegravir use, and retention-in-care and viral suppression, and c) the effect of dolutegravir use on HIV treatment outcomes among people receiving concurrent tuberculosis treatment.

## METHODS

### Study design and setting

We conducted a cohort study using de-identified, routinely collected data from 59 public sector, primary care clinics run by the eThekwini Municipality Health Unit, in the province of KwaZulu-Natal, South Africa. ART is provided free of charge in accordance with South African National Department of Health guidelines,^12^ which recommended dolutegravir from December 1, 2019. Initially, ART initiations were prioritised, and women of child-bearing potential required to sign an ‘acknowledgement of risk’ form. On February 24, 2020, eligibility was expanded to include people already receiving first-line ART, with a risk-benefit discussion replacing the signed risk form for women of child-bearing potential.^13^ In June 2021, the risk-benefit discussion for women was removed, and dolutegravir became the preferred first-line ART regimen.^14^ For PLHIV with tuberculosis co-infection, dolutegravir was only recommended after completing tuberculosis treatment. Viral load testing was recommended annually, and transition to first-line dolutegravir was only recommended if people had a suppressed viral load of <50 copies/mL in the previous six months, or consecutive viral loads between 50–999 copies/mL.^12^

### Participants

We analysed two mutually exclusive groups; the ‘initiator cohort’, and the ‘transition cohort’. For the initiator cohort, we evaluated dolutegravir use by gender among all PLHIV aged ≥15 years and newly initiating first-line ART (irrespective of NRTI backbone) at participating clinics, between December 1, 2019 and February 28, 2022. We excluded people with known previous ART exposure, as South African guidelines recommend re-initiating the previous regimen, which would often not be dolutegravir-based. We then analysed outcomes among those who initiated ART before December 1, 2020 and therefore had at least 12 months of follow-up time plus 90 days to assess retention-in-care before the data cut on February 28^th^, 2022. We assessed viral load outcomes only among those who were retained-in-care at 12 months.

For the transition cohort, we assessed transition to first-line dolutegravir by gender among PLHIV aged ≥15 years who were in care and receiving non-dolutegravir first-line ART at participating clinics on December 1^st^, 2019. We excluded those with viral load ≥1000 copies/mL in the previous 12-months, as they would not have been eligible for transition to first-line dolutegravir. We then analysed outcomes among a subset who had initiated dolutegravir before December 1, 2020, compared to people who had not initiated dolutegravir at the same timepoint, matched 1:1 using propensity score matching (appendix pp 1–2).

### Data sources and data management

For patients initiating and receiving ART in the South African public sector, data on demographics, clinical status, ART and clinic visits are routinely recorded in the TIER.net^15^ electronic register. TIER.net data from participating clinics was collated and de-identified by the South African National Department of Health’s TB/HIV Information Systems (THIS -www.tbhivinfosys.org.za/) before being extracted for analysis.

We used demographic and clinical variables recorded at ART initiation or during follow-up.

#### Outcomes

We defined first-line dolutegravir use from the ART initiation regimen (initiator cohort) or the date of first use of dolutegravir in a first-line regimen (transition cohort, see appendix pp1 for full definition). We defined retention-in-care as not being recorded in TIER.net as deceased, lost-to-follow-up (defined as 90 days late for a visit by the South African ART programme, with date of last visit used as date of loss-to-follow-up) or ‘transferred out’ to another clinic (as we could not access or link to data at other clinics to establish retention-in-care). We used viral suppression <50 copies/ml, as per clinical trials^16–18^ and South African guidelines. In this programmatic setting, not all patients had annual viral loads. Therefore, in both cohorts we included viral loads five to 18 months after baseline, and used the result closest to 12 months.

### Statistical analysis

Among the initiator cohort, we first used univariable Poisson regression models with robust standard errors to estimate relative risks for the association between gender, and the outcome of dolutegravir initiation. We also conducted sensitivity analyses, defined a priori, to assess whether the effect of gender on dolutegravir use was modified by age at ART initiation, and by time. We then used univariable and multivariable Poisson regression models with robust standard errors to assess the association between being initiated on dolutegravir, and 12-month retention-in-care, and viral suppression. To assess whether tuberculosis treatment impacted on viral suppression with dolutegravir, we did a sensitivity analyses, defined a priori, with an interaction term between dolutegravir use and tuberculosis status.

Among the transition cohort, we used univariable Cox proportional hazards models to assess the association between gender and time to transition to a dolutegravir-based first-line regimen. We started follow-up time from December 1, 2019, and censored people at date of loss-to-follow-up, death, transfer to another clinic, or switch to second-line ART. We also censored people with a viral load of >1000 copies/mL during follow-up, as they would not have been eligible for immediate transition to first-line dolutegravir.

Comparing outcomes between people transitioned and not transitioned to dolutegravir is complicated by the lack of a clear baseline time in those whose treatment remains unchanged, and potential underlying differences between the two groups. We addressed these issues by emulating a ‘target trial’, in which each person transitioned to dolutegravir was matched to a control who had not yet been transitioned at the same timepoint.^19^ To increase comparability between the two groups, we matched participants 1:1 using a time-dependent propensity score of the log-hazard of transition to dolutegravir,^20,21^ with direct matching by time since most recent viral load, and whether the participant was in a differentiated ART delivery programme. Individuals could only be matched once, and we only matched participants transitioned to dolutegravir before December 1^st^, 2020, to allow 12 months of follow-up time. Further details are provided in the appendix pp 1–2. We then used multivariable Poisson regression models to compare the outcomes of 12-month retention-in-care and viral suppression between people transitioned to dolutegravir, and their matched controls.

In both the initiation and transition cohort, the primary viral load analysis was an intention-to-treat analysis, with a secondary ‘as-treated’ analysis that excluded people who changed ART regimen after baseline. Dolutegravir rollout and clinical outcomes may have varied by clinic, and we accounted for this using robust standard errors. We did not adjust for multiple comparisons. We present both risk ratios and risk differences to demonstrate both the relative strength of an association, and the absolute difference. We included a specific category for missing baseline CD4 count data. We analysed data using R 4.2.0 (R Foundation for Statistical Computing, Vienna, Austria) and STATA 14.0 (StataCorp, Texas, USA).

### Ethical approval

This work was approved by the University of Kwazulu-Natal Biomedical Research Ethics Committee (BE646/17), the KwaZulu-Natal Provincial Health Research Ethics Committee (KZ_201807_021), the THIS Data Request Committee, and the eThekwini Municipality Health Unit, with a waiver for informed consent for analysis of de-identified, routinely collected data.

### Role of the funding source

The funders had no role in the study design, data collection, analysis, interpretation, writing of the paper, or the decision to submit for publication.

## RESULTS

**Figure 1:**
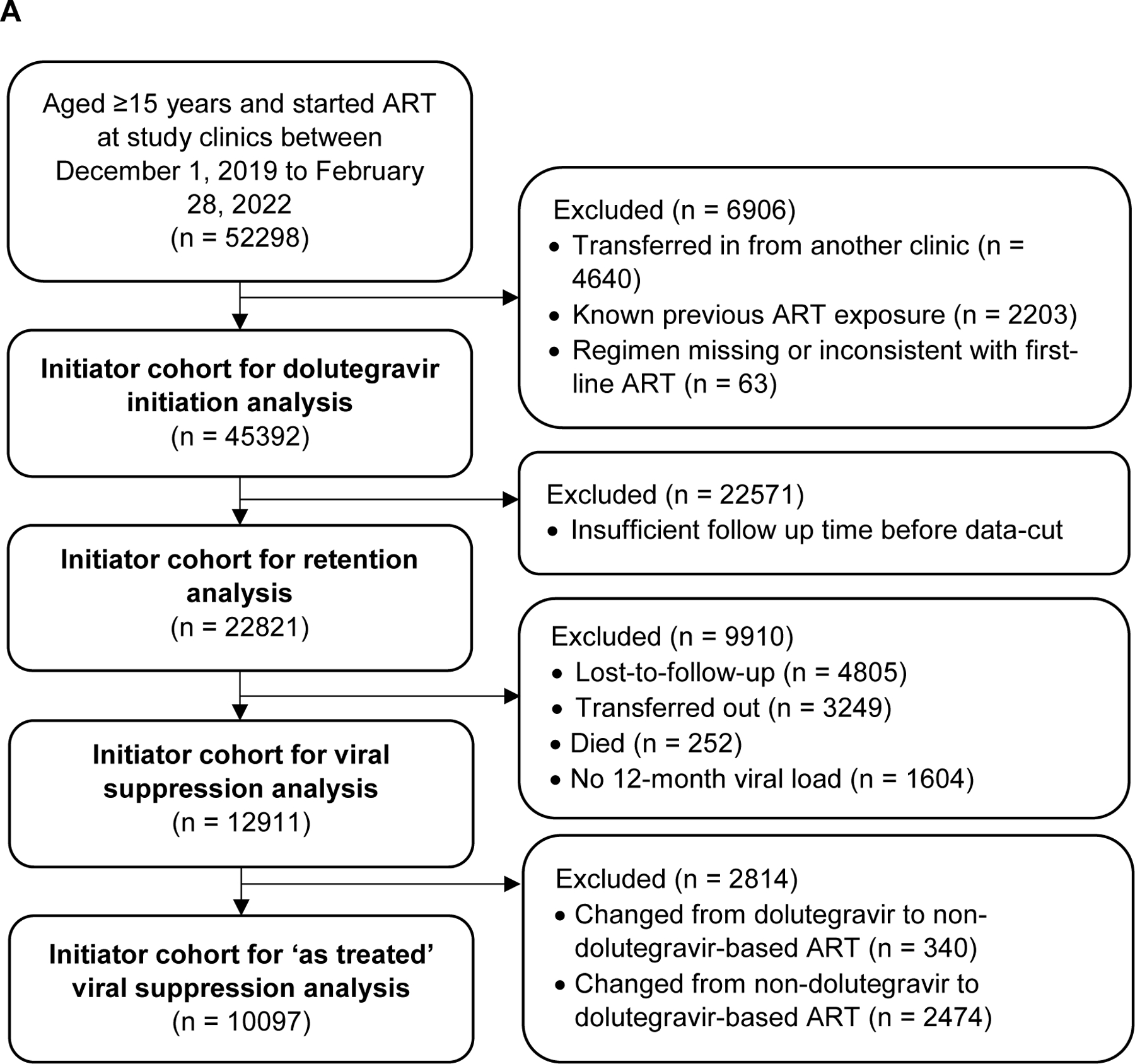

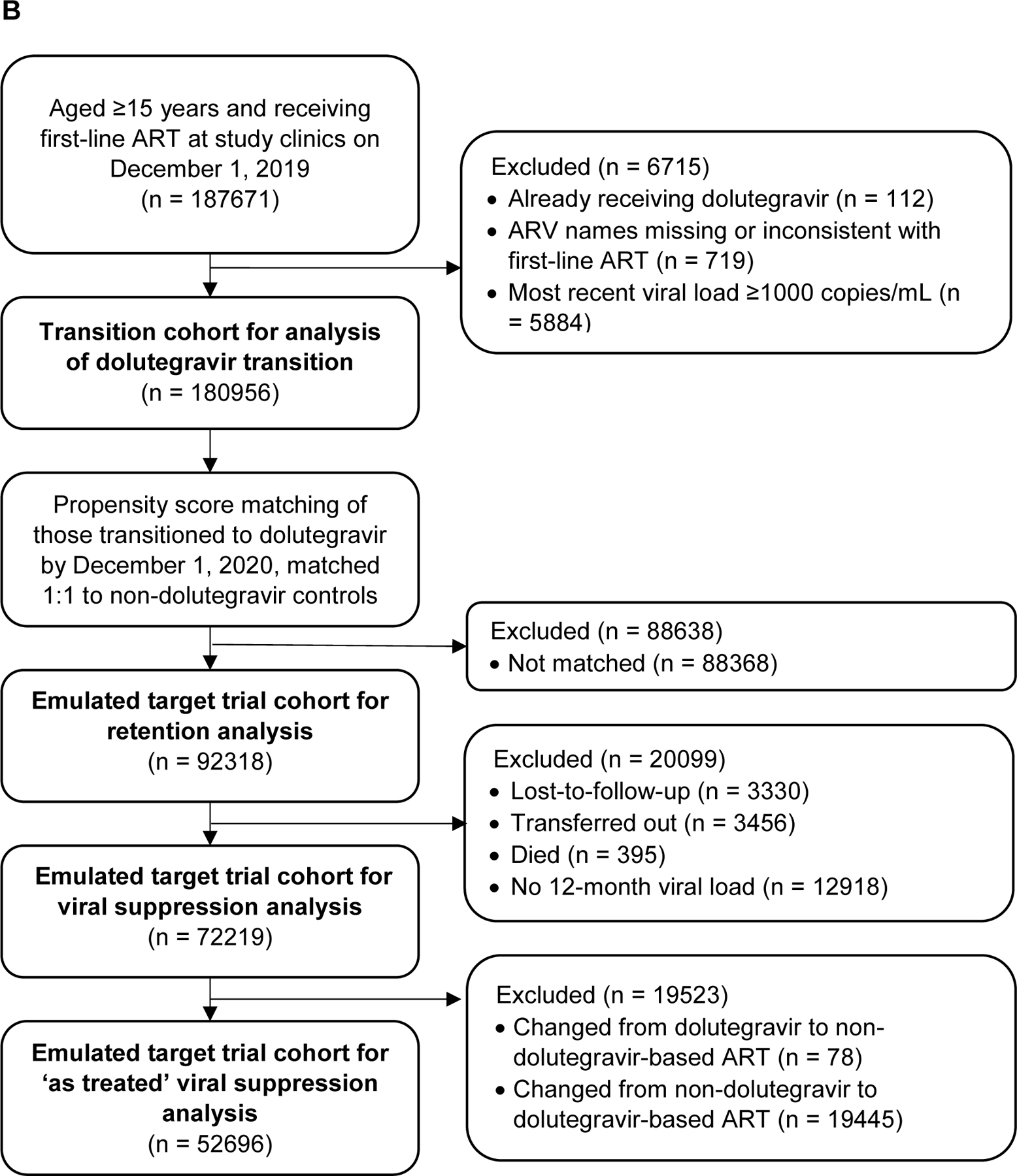
Flow diagram of A) initiation and B)transition cohort at 59 primary care clinics in South Africa

In the initiator cohort, between December 1^st^, 2019 and February 28^th^, 2022, 45,392 people were initiated on ART (Figure 1A). 23,945 (52·8%) were non-pregnant women, 4,780 (10·5%) were pregnant women, and 16,667 (36·7%) were men (Table 1). Median age was 31·0 years (IQR 26·0, 38·0) and 2401 (5·3%) were receiving TB treatment at time of ART initiation.

31,264 (69·9%) people were initiated on dolutegravir, 14,102 (31·1%) on efavirenz, and 26 (0·1%) on nevirapine. Dolutegravir use increased over time; between December 1, 2019 to February 29, 2020, 19·2% were initiated on dolutegravir-based ART, compared to 96·6% between December 1, 2021 and February 28, 2022 (Table 1). Over the study period, 46·9% of pregnant women, 63·9% of non-pregnant women and 82·3% of men were initiated on dolutegravir. In a univariable Poisson regression model, pregnant women (risk ratio [RR] 0·57 [95% confidence interval 0·49, 0·66], risk difference [RD] −35·4% [−42·3, −28·5]) non-pregnant women (RR 0·78 [0·74, 0·82], RD −18·4% [−21·6, −15·2]) were less likely to be initiated on dolutegravir than men (Table 1).

The effect of gender was strongest among people aged 15–24 years (non-pregnant women versus men RR 0·73 [0·69, 0·77]), and decreased with older age, with no difference between men and women in the ≥55 year-old group (RR 0·97 [0·90, 1·03], Wald test for interaction p <0·0001, Table S2 appendix pp 2). Early in the rollout women were much less likely to receive dolutegravir than men (December 1, 2019 to February 29, 2020: non-pregnant women RR 0·29 [0·23, 0·38], pregnant women 0·25 [0·12, 0·53], compared to men, Table S2 appendix pp 2), but this difference declined and disappeared by September 1, 2021 to November 30, 2021 for both non-pregnant (RR 1·00 [0·98, 1·03]) and pregnant women (RR 0·98 [0·94, 1·02], Wald test for interaction p <0·0001).

22,821 people initiated ART before December 1, 2020, and had the opportunity to complete 12 months of follow-up, plus 90 days to assess retention, before the data cut. Of those who were initiated on a dolutegravir-based regimen, 476/10868 (4·4%) were changed to efavirenz (n=462) or another non-dolutegravir based regimen (n = 14), after a median 120 days (66, 201) from ART initiation. Of those initiated on a non-dolutegravir regimen, 2944/11953 (24·6%) were changed to dolutegravir, after a median of 191 days (98, 252).

By 12 months, in the dolutegravir group 7108 (65·4%) were retained-in-care, 1391 (12·8%) had transferred to another clinic, 2233 (20·5%) were lost-to-follow-up, and 136 (12·8%) were known to have died, compared to 7407 (62·0%) retained-in-care, 1858 (15·5%) transferred, 2572 (21·5%) lost-to-follow-up and 116 (9·7%) died in the non-dolutegravir group. Overall median time to loss-to-follow-up was 35 days (0–156); 1895 (39·4%) were not seen again after ART initiation. In a Poisson model adjusted for age, gender (including pregnancy), time, tuberculosis status and initiation CD4 count, people initiated on dolutegravir were more likely to be retained-in-care compared to those initiated on non-dolutegravir based regimens (adjusted risk ratio [aRR] 1·09 [1·04, 1·14], aRD 5·2% [2·2, 8·4], Table 2). In post-hoc sensitivity analysis including transfers to another clinic as retained-in-care, the association between dolutegravir and retention was attenuated (aRR 1·03 [0·99, 1·08], aRD 2·4% [−0·9, 5·7]).

Of those retained-in-care at 12 months, 12,911/14,515 (88·9%) had a documented viral load result after a median 365 (347, 380) days from initiation. Of these, 10,616 (82·2%) were supressed <50 copies/mL. In a multivariable model adjusted for the same variables as the retention model, people initiated on dolutegravir had better viral suppression compared to non-dolutegravir regimens (aRR 1·04 [1·01, 1·06], aRD 3·1% [1·2, 5·1], Table 3). In ‘as treated’ analyses, excluding people who had a change in ART from or to dolutegravir after ART initiation, the association between dolutegravir and viral suppression was stronger (aRR 1·09 [1·05, 1·12], aRD 6·8% [4·3, 9·4]) Table S3 appendix pp3).

Among people with tuberculosis, 316/774 (40·8%) were initiated on dolutegravir. In this group, the association between dolutegravir and viral suppression was stronger (aRR 1·14 [1·07, 1·22]) than among those without tuberculosis (aRR 1·03 [1·01, 1·06], Wald test for interaction p = 0·006, Table S4 appendix pp3).

For the transition cohort, on December 1^st^, 2019, 180,956 people were receiving non-dolutegravir based first-line ART at the study clinics (Figure 1B), of whom 124,168 (68·6%) were women (Table 4). Median age was 38 (32, 45) years, and median time on ART was 3·9 (2·0, 6·4) years, with the majority receiving efavirenz (98·7%) and tenofovir (98·4%).

Participants were followed up to endpoint or censoring date for a median 319 days (IQR 205, 646), totalling 206,050 person-years at risk. By February 28, 2022, 121,174 (67·0%) had transitioned to first-line dolutegravir at a median of 283 (IQR 203, 526) days. 27,702 (15·3%) remained in care and continued receiving non-dolutegravir-based first-line ART. 9404 (5·2%) had a viral load ≥ 1000 copies/mL, 657 (0·4%) had been switched to second-line ART, 12,074 (6·7%) transferred care to another clinic, 9,127 (5·0%) were lost-to-follow-up and 818 (0·5%) were known to have died. The rate of dolutegravir transition peaked between June 1, 2020 and August 31, 2020 (1118·6 [1107·0 to 1130.4] per 1000 person-years), and was lowest between December 1, 2020 and February 28, 2021 (345·3 [337·1 to 353·7] per 1000 person-years (Table S5 appendix pp 3, Figure 2).

In a univariable Cox regression model the hazard of being transitioned to dolutegravir was lower in women than in men (hazard ratio [HR] 0·56 [0·56, 0·57], Table 4). The effect of gender on dolutegravir transition was largest among younger age groups (15–24 years, HR 0·50 [0·46, 0·53], ≥55 years, HR 0·93 [0·90, 0·97], Table S6 appendix pp 4, likelihood ratio test for interaction p <0·0001). When including an interaction term between gender and time, the hazard of initiating dolutegravir was lower in women compared to men earlier in the rollout (December 1, 2019 to February 29, 2020, HR 0·37 [0·34, 0·40]), but converged as the rollout progressed, and became higher in women than men by September 1, 2021 to November 30, 2021 (HR 1·09 [1·04, 1·15], likelihood ratio test for interaction p < 0·0001, Table S6 appendix pp 4).

75,223 had been transitioned to dolutegravir before December 1, 2020. After propensity score matching, 46,159 people who transitioned to dolutegravir were matched with 46,159 controls who had not yet been transitioned at the same timepoint, and were included in the target trial. Baseline characteristics were well balanced between the two groups (Table S7 appendix pp 4). In the dolutegravir group, 88/46,159 (0·2%) people subsequently changed back to a non-dolutegravir first-line regimen, at a median of 211 days (IQR 116, 308) from baseline. In the matched controls, 23,620/46,159 (51·2%) subsequently transitioned to dolutegravir at a median of 154 days (73, 253) from baseline. By 12 months, in the dolutegravir group 43,178 (93·5%) were retained-in-care, 1388 (3·0%) had transferred out, 1460 (3·2%) were lost-to-follow-up and 133 (0·3%) were known to have died, compared to 41,959 (90·9%) retained-in-care, 2068 (4·5%) transferred, 1870 (4·1%) lost-to-follow-up and 262 (0·6%) died in the non-dolutegravir group. The likelihood of retention in care was higher among the dolutegravir group versus matched controls (aRR 1·03 [1·02, 1·03], RD 2·5%, 2·1, 2·9), but was partially attenuated in post-hoc sensitivity analysis where transfers out were included as retained in care (aRR 1·01 [1·00, 1·01], RD 1·1% [0·7, 1·5]). Among those retained-in-care, 72,219 (84·8%) had a viral load result after median 336 days (266, 422) from baseline. In the dolutegravir group, 33,423 (90·5%) were suppressed at <50 copies/mL, compared to 31,648 (89·7%) in matched controls (aRR 1·01 [1·00, 1·02], RD 0·8% [0·3, 1·4]). In sensitivity analysis with an interaction term between dolutegravir use and baseline viral load, dolutegravir was only associated with improved viral suppression among people with baseline viral load ≥200 copies/mL (e.g. 200–399 copies/mL, aRR 1.15 [1.05, 1.26], 400–999 copies/mL, aRR 1.25 [1.13, 1.38], Table S8 appendix pp 5). In ‘as treated’ analysis, the association between dolutegravir and viral suppression was slightly stronger (aRR 1·03 [1·02, 1·04], RD 2·3% [1·4, 3·2], Table S8 appendix pp 5).

## DISCUSSION

In this large cohort study from 59 South African clinics, women were less likely to receive dolutegravir compared to men, with the effect strongest early in the rollout and among younger women. In both people initiating and already receiving first-line ART, dolutegravir use was associated with better 12-month retention-in-care and viral suppression. The association between dolutegravir use and viral suppression was stronger among people receiving concurrent tuberculosis treatment when initiating ART, and among people transitioned to dolutegravir with most recent viral load at baseline ≥200 copies/mL.

We provide longer term data compared to studies from earlier in the dolutegravir rollout, which showed that initially younger women were less likely to receive dolutegravir.^7–9^ A multi-site study of 134,672 PLHIV from 11 countries up to March 2020 (eight months after the WHO recommended dolutegravir for all PLHIV), found that in people aged 16–49, dolutegravir use was lower among women compared to men, but longer term trends were not assessed.^7^ In our study, younger women were less likely to receive dolutegravir up to two years after WHO recommended dolutegravir for all. This difference decreased over time and disappeared shortly after South African guidelines recommended dolutegravir for all in June 2021, with the hazard of transition to dolutegravir becoming higher among women compared to men in the subsequent months; a likely ‘catch-up’ effect. However, by the end of the follow-up period, women remained overall less likely to be on dolutegravir than men, and 15% of people remained on non-dolutegravir first-line ART.

Retention-in-care at 12-months was low at 63·6% among people newly initiating ART in our cohort, with a higher proportion (14·2%) transferring to other clinics than seen in a previous study (6·1%),^22^ which may reflect mobility due to COVID-19. Our finding that first-line dolutegravir was associated with better retention-in-care may be due to increased tolerability, which could be particularly important early in treatment. This is similar to findings from clinical trials, in which the superior efficacy of dolutegravir has largely been driven by reduced discontinuations from adverse events.^16–18^ However, better retention was partly driven by more clinic transfers among people receiving efavirenz, reasons for which are not clear, but could represent people seeking more tolerable ART and better treatment at another clinic. We also found better 12-month viral suppression with dolutegravir, particularly in people initiating ART, with the risk difference within the range seen in two clinical trials in Africa.^16,18^ Among those transitioning from other first-line ART regimens (a group in which no clinical trials have been conducted), we only found improved viral suppression with dolutegravir among those with most recent viral loads ≥200 copies/mL. This could be due to HIV drug resistance against efavirenz among people with low level viraemia, better efficacy of dolutegravir at lower levels of adherence, or improved adherence due to better tolerability of dolutegravir.

There is little data from non-trial settings directly comparing outcomes between dolutegravir and other regimens, particularly in Africa. Public health data from Brazil showed good safety outcomes,^23^ and better 12-month viral suppression among people initiated on dolutegravir compared to efavirenz,^24^ but the proportion on dolutegravir was low (10·5%), and there was no assessment of retention-in-care. Studies from Malawi, Lesotho, and Uganda suggest low levels of dolutegravir HIV drug resistance mutations, and high levels of viral suppression, among people transitioned to dolutegravir, but do not compare results with people remaining on non-dolutegravir regimens.^25–27^ A retrospective cohort study of 3,108 people from four African countries found that people transitioned to dolutegravir had better viral suppression compared to those who remained on the same first or second-line regimen.^10^ However, analyses such as this are susceptible to bias because it is difficult to choose an appropriate baseline time zero in the control group whose treatment remains unchanged, resulting in baseline timepoints and viral load schedules differing between the two groups, and potential for immortal time bias. We emulated a target trial to overcome this, and present the largest analysis comparing outcomes after transition to dolutegravir among people on first-line ART, who are the largest group of people who will use dolutegravir globally.

South African guidelines recommend efavirenz rather than dolutegravir among people initiating ART who are receiving rifampicin containing tuberculosis treatment. However, we found that over 50% (n=1305) did receive dolutegravir, and among this group, the beneficial effect of dolutegravir on viral suppression was even stronger. The INSPIRING trial demonstrated good tolerability and acceptable viral suppression among people receiving tuberculosis treatment who were given double dose dolutegravir.^11^ While the extent of dolutegravir double dosing is not recorded in our data, our findings provide re-assurance that co-treating TB-HIV co-infection did not compromise HIV outcomes in a high TB burden programmatic setting. Our findings are supported by a smaller cohort study including 465 people receiving dolutegravir co-treatment alongside tuberculosis treatment, which found better viral suppression compared to people receiving co-treatment with efavirenz.^28^

Our study is the largest to evaluate dolutegravir uptake and compare subsequent treatment outcomes against non-dolutegravir based regimens in a public health programme. We used data from routine public sector clinics, which provide care according to South African Department of Health guidelines, and used programmatic outcome definitions,^12^ making our findings more generalisable to other public sector settings (although our clinics were limited to one urban district). We directly compared both retention-in-care and viral suppression between dolutegravir and non-dolutegravir regimens, with precise estimates due to the large sample size. Our use of an emulated target trial in the transition cohort, with propensity score matching to balance the dolutegravir and non-dolutegravir groups, should increase comparability, although we cannot rule out residual unmeasured confounding. We are likely to have overestimated loss-to-follow-up as mortality and ‘silent transfers’ to other clinics are underestimated in TIER.net.^29^ As we used routinely collected data, we were unable to search for silent transfers to other clinics, and did not have consent to search for deaths on the national registry. We assessed 12-month treatment outcomes, and further work will be required to assess if outcomes remain similar after longer follow-up. We were unable to assess the dose of dolutegravir used in people with tuberculosis, HIV drug resistance and adverse events such as weight gain, as these are not recorded in TIER.net.

Our findings are important as they demonstrate the extent to which women were excluded from the early dolutegravir rollout. They also provide reassurance that in programmatic settings, dolutegravir is associated with similar or better outcomes than efavirenz, reflecting findings from clinical trials. While improvements in retention-in-care and viral suppression with dolutegravir were modest, incremental gains are important in reaching the 95–95-95 targets. However, overall loss-to-follow-up of 20% by 12 months among people newly initiating ART demonstrates that early retention-in-care remains a key challenge for HIV programmes, and this could limit the potential benefit of improved ART regimens. Strategies to identify and support people at risk of loss to follow up are therefore needed. Our findings support ongoing efforts to continue the transition to dolutegravir, and to remove restrictions on dolutegravir use among people being treated for tuberculosis. Efforts should particularly focus on ensuring that women receive updated safety information and are provided the opportunity to use dolutegravir without restrictions. More broadly, strategies to introduce newer antiretrovirals at scale should ensure that the necessary safety evidence is been generated as quickly as possible prior to rollout, and that pregnant women are included in drug trials where possible. Further research is needed in non-trial settings to assess reasons for not transitioning to dolutegravir,^30^ adverse events such as weight gain and metabolic consequences (which seem to disproportionately affect women),^31^ the impact of transition to dolutegravir among people with viraemia ≥1000 copies/mL,^32^ and the use of dolutegravir in second-line regimens.^33^

In conclusion, we found that young women were less likely to receive dolutegravir until September 2021, and that people receiving dolutegravir had better retention-in-care and viral suppression compared to efavirenz. Efforts to transition to dolutegravir should continue.

## Supplementary Material

3

## Figures and Tables

**Figure 2:**
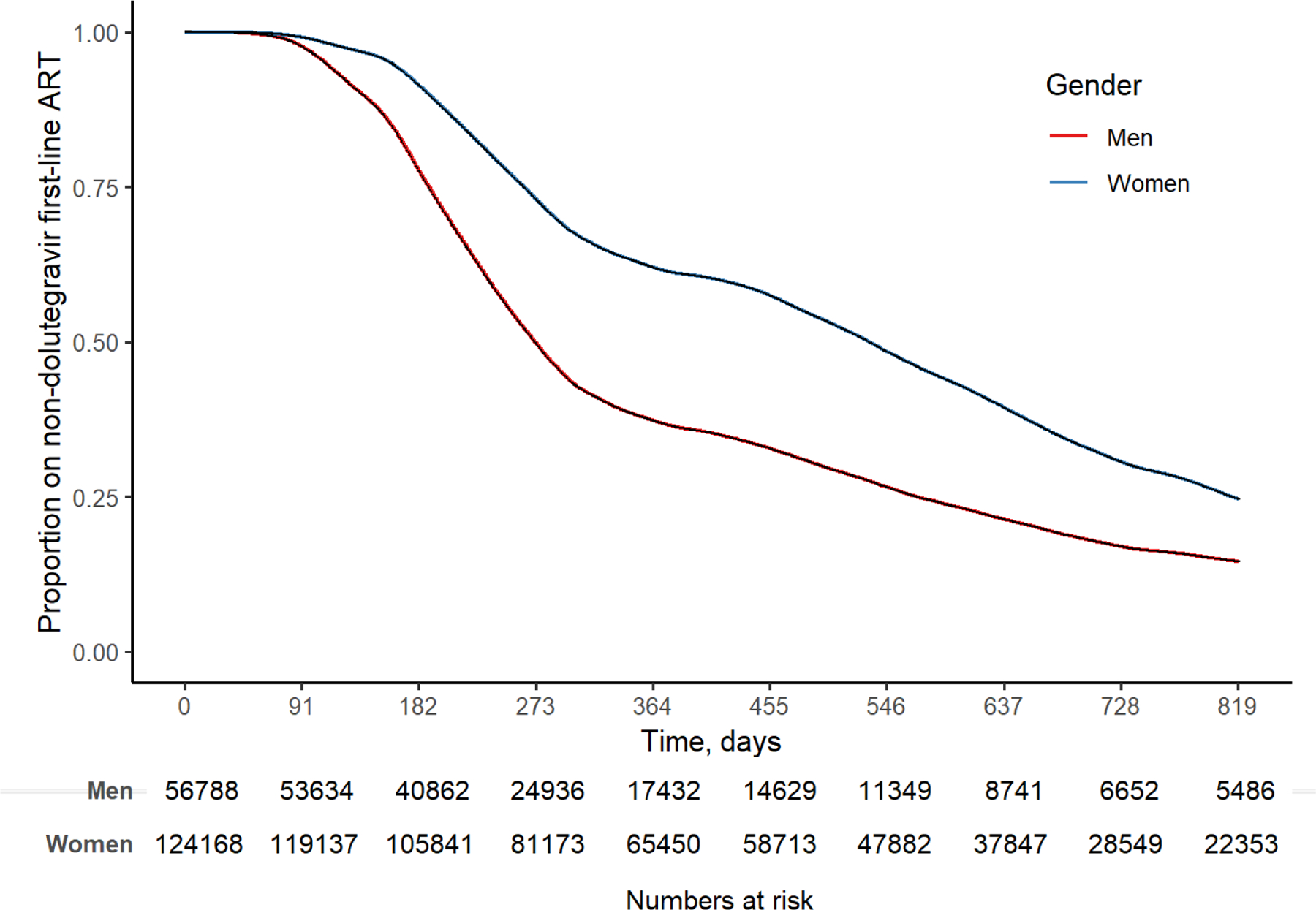
Kaplan Meier curve of transition to dolutegravir among people already receiving non-dolutegravir based first-line ART in December 2019

**Table 1:** Univariable Poisson regression models of baseline characteristics associated with being initiated on dolutegravir-based first line ART versus non-dolutegravir ART, n = 45,392

Variable	Level	Total (column %)	Non-dolutegravir regimen, n (row %)	Dolutegravir regimen, n (row %)	RR
Gender	Male	16667 (36·7)	2948 (17·7)	13719 (82·3)	1
	Female, not pregnant	23956 (52·8)	8650 (36·1)	15306 (63·9)	0·78 (0·74–0·82)
	Female, pregnant	4769 (10·5)	2530 (53·1)	2239 (46·9)	0·57 (0·49–0·66)
Age (years)	55+	1170 (2·6)	277 (23·7)	893 (76·3)	1
	45–54	3823 (8·4)	862 (22·5)	2961 (77·5)	1·01 (0·98–1·05)
	35–44	11833 (26·1)	3127 (26·4)	8706 (73·6)	0·96 (0·93–1·00)
	25–34	20274 (44·7)	6690 (33·0)	13584 (67·0)	0·88 (0·84–0·92)
	15–24	8292 (18·3)	3172 (38·3)	5120 (61·7)	0·81 (0·76–0·86)
Initiation time period	Dec 19 - Feb 20	7174 (15·8)	5797 (80·8)	1377 (19·2)	1
	Mar 20 - May 20	5582 (12·3)	2953 (52·9)	2629 (47·1)	2·45 (2·03–2·96)
	Jun 20 - Aug 20	4940 (10·9)	1730 (35·0)	3210 (65·0)	3·39 (2·74–4·19)
	Sep 20 - Nov 20	5125 (11·3)	1473 (28·7)	3652 (71·3)	3·71 (2·99–4·60)
	Dec 20 - Feb 21	4663 (10·3)	936 (20·1)	3727 (79·9)	4·16 (3·34–5·20)
	Mar 21 - May 21	5668 (12·5)	602 (10·6)	5066 (89·4)	4·66 (3·72–5·83)
	Jun 21 - Aug 21	4216 (9·3)	321 (7·6)	3895 (92·4)	4·81 (3·85–6·02)
	Sep 21 - Nov 21	4063 (9·0)	180 (4·4)	3883 (95·6)	4·98 (3·97–6·24)
	Dec 21 - Feb 22	3961 (8·7)	136 (3·4)	3825 (96·6)	5·03 (4·03–6·28)
TB at ART initiation	No TB	42991 (94·7)	13032 (30·3)	29959 (69·7)	1
	Known TB	2401 (5·3)	1096 (45·6)	1305 (54·4)	0·78 (0·70–0·87)
Initiation CD4 count (cells/µL)	<200	7038 (15·5)	2090 (29·7)	4948 (70·3)	1
	200–349	7543 (16·6)	2381 (31·6)	5162 (68·4)	0·97 (0·95–1·00)
	350–499	6704 (14·8)	2187 (32·6)	4517 (67·4)	0·96 (0·93–0·98)
	>=500	11020 (24·3)	3670 (33·3)	7350 (66·7)	0·95 (0·92–0·98)
	Missing	13087 (28·8)	3800 (29·0)	9287 (71·0)	1·01 (0·94–1·09)

**Table 2:** Univariable and multivariable Poisson regression models of factors associated with retention-in-care in people initiating dolutegravir and non-dolutegravir-based first line ART

Retention-in-care (n = 22,821)				
Variable	Levels	Retained-in-care	RR	aRR*
ART regimen	Non-DTG regimen	7407/11953 (62·0)	1	1
	DTG regimen	7108/10868 (65·4)	1·06 (1·02–1·10)	1·09 (1·04–1·14)^†^
Gender	Male	5432/8337 (65·2)	1	1
	Female, not pregnant	7518/11985 (62·7)	0·96 (0·94–0·99)	1·04 (1·02–1·07)
	Female, pregnant	1565/2499 (62·6)	0·96 (0·91–1·02)	1·10 (1·02–1·18)
Age (years)	55+	415/599 (69·3)	1	1
	45–54	1368/1872 (73·1)	1·05 (1·00–1·11)	1·05 (0·99–1·11)
	35–44	3945/5767 (68·4)	0·99 (0·94–1·04)	0·99 (0·94–1·04)
	25–34	6461/10377 (62·3)	0·90 (0·86–0·94)	0·91 (0·86–0·95)
	15–24	2326/4206 (55·3)	0·80 (0·75–0·85)	0·81 (0·76–0·86)
Initiation time period	Dec 19 - Feb 20	4624/7174 (64·5)	1	1
	Mar 20 - May 20	3637/5582 (65·2)	1·01 (0·98–1·05)	0·98 (0·95–1·02)
	Jun 20 - Aug 20	3083/4940 (62·4)	0·97 (0·93–1·00)	0·94 (0·89–0·99)
	Sep 20 - Nov 20	3171/5125 (61·9)	0·96 (0·92–1·00)	0·93 (0·88–0·98)
TB at ART initiation	No TB	13646/21660 (63·0)	1	1
	Known TB	869/1161 (74·8)	1·19 (1·14–1·23)	1·17 (1·13–1·22)
Initiation CD4 count (cells/µL)	<200	2645/3709 (71·3)	1	1
	200–349	2724/3979 (68·5)	0·96 (0·93–0·99)	0·99 (0·96–1·03)
	350–499	2291/3459 (66·2)	0·93 (0·90–0·96)	0·98 (0·94–1·01)
	>=500	3539/5523 (64·1)	0·90 (0·87–0·93)	0·95 (0·91–0·98)
	Missing	3316/6151 (53·9)	0·76 (0·69–0·83)	0·78 (0·72–0·86)

* The primary exposure effect (dolutegravir use) is adjusted for all other variables in the model as potential confounders. Unlike the primary exposure effect, the presented adjusted risk ratios for potential confounding variables should not be interpreted as the effect of the confounding variable on the outcome.

† Estimated risk difference (RD) 5·2%, 95%CI 2·2 to 8·4.

**Table 3:** Univariable and multivariable Poisson regression models of factors associated with viral suppression in people initiating dolutegravir and non-dolutegravir-based first line ART

Viral suppression (n = 12,911)				
Variable	Levels	Viral suppression	RR	aRR*
ART regimen	Non-DTG regimen	5327/6541 (81·4)	1	1
	DTG regimen	5289/6370 (83·0)	1·02 (1·00–1·04)	1·04 (1·01–1·06)^†^
Gender	Male	3808/4796 (79·4)	1	1
	Female, not pregnant	5685/6771 (84·0)	1·06 (1·04–1·07)	1·06 (1·04–1·07)
	Female, pregnant	1123/1344 (83·6)	1·05 (1·02–1·08)	1·06 (1·02–1·09)
Age (years)	55+	319/379 (84·2)	1	1
	45–54	1036/1248 (83·0)	0·99 (0·94–1·04)	0·99 (0·94–1·04)
	35–44	2902/3556 (81·6)	0·97 (0·93–1·01)	0·98 (0·93–1·02)
	25–34	4707/5708 (82·5)	0·98 (0·93–1·03)	0·97 (0·92–1·02)
	15–24	1652/2020 (81·8)	0·97 (0·93–1·02)	0·94 (0·90–0·99)
Initiation time period	Dec 19 - Feb 20	3324/4117 (80·7)	1	1
	Mar 20 - May 20	2746/3288 (83·5)	1·03 (1·01–1·06)	1·03 (1·00–1·05)
	Jun 20 - Aug 20	2325/2740 (84·9)	1·05 (1·03–1·08)	1·03 (1·00–1·06)
	Sep 20 - Nov 20	2221/2766 (80·3)	0·99 (0·97–1·02)	0·98 (0·95–1·00)
TB at ART initiation	No TB	10032/12137 (82·7)	1	1
	Known TB	584/774 (75·5)	0·91 (0·88–0·95)	0·96 (0·93–1·00)
Initiation CD4 count (cells/µL)	<200	1786/2426 (73·6)	1	1
	200–349	2011/2473 (81·3)	1·10 (1·07–1·14)	1·10 (1·07–1·14)
	350–499	1734/2052 (84·5)	1·15 (1·11–1·19)	1·14 (1·11–1·18)
	>=500	2798/3177 (88·1)	1·20 (1·17–1·23)	1·19 (1·15–1·22)
	Missing	2287/2783 (82·2)	1·12 (1·08–1·15)	1·11 (1·08–1·15)

* The primary exposure effect (dolutegravir use) is adjusted for all other variables in the model as potential confounders. Unlike the primary exposure effect, the presented adjusted risk ratios for potential confounding variables should not be interpreted as the effect of the confounding variable on the outcome.

† Estimated RD 3·1%, 95%CI 1·2 to 5·1.

**Table 4:** Univariable Cox regression models of baseline characteristics associated with transitioning to dolutegravir-based first line ART, N = 180,956

Variable	Levels	Total, n (column %)	Transition to dolutegravir events	Time, person-years	Rate per 100 person-years (95% CI)	HR
Gender	Male	56788 (31·4)	41967	52094	805·6 (797·9–813·3)	1
	Female	124168 (68·6)	79207	153937	514·5 (511·0–518·1)	0·56 (0·56–0·57)
Baseline age (cat.),	55+	14010 (7·7)	10075	14302	704·4 (690·7–718·3)	1
years	45–54	34223 (18·9)	25720	35322	728·2 (719·3–737·1)	1·04 (1·01–1·06)
	35–44	67118 (37·1)	46514	77154	602·9 (597·4–608·4)	0·82 (0·80–0·84)
	25–34	55944 (30·9)	33822	67823	498·7 (493·4–504·0)	0·66 (0·64–0·67)
	15–24	9661 (5·3)	5043	11431	441·2 (429·1–453·5)	0·56 (0·54–0·58)
Baseline time on ART, years	Median (IQR)	3·9 (2·0 to 6·4)	-	-	-	1·01 (1·01–1·01)
Baseline most recent	>=500	70180 (38·8)	47141	83106	567·2 (562·1–572·4)	1
CD4 count (cat.),	350–499	39875 (22·0)	27399	45353	604·1 (597·0–611·3)	1·10 (1·08–1·11)
cells/µL	200–349	34093 (18·8)	23200	37544	617·9 (610·0–625·9)	1·14 (1·12–1·15)
	<200	21477 (11·9)	14139	22859	618·5 (608·4–628·8)	1·17 (1·14–1·19)
	Missing	15331 (8·5)	9295	17170	541·3 (530·4–552·5)	0·98 (0·96–1.00)
TB during follow-up	No	178426 (98·6)	121008	205179	589·8 (586·4–593·1)	1
	Yes	2530 (1·4)	166	853	194·7 (166·2–226·7)	0·41 (0·35–0·48)
Pregnancy during	No	116628 (64·5)	120826	204581	590·6 (587·3–593·9)	1
follow-up	Yes	7540 (4·2)	348	1450	239·9 (215·4–266·5)	0·40 (0·36–0·44)
In CCMDD during	No	85523 (47·3)	79942	122844	650·8 (646·3–655·3)	1
follow-up?	Yes	95433 (52·7)	41232	83187	495·7 (490·9–500·5)	0·68 (0·67–0·69)

CCMDD; the Centralised Chronic Medication Dispensing & Distribution programme (a differentiated ART delivery programme)

## RESEARCH IN CONTEXT

### Evidence before this study

We searched PubMed with no language restrictions on October 5, 2022, with the terms [dolutegravir AND (rollout OR implementation OR outcomes) AND Africa] and identified additional studies using hand searches of reference lists and citing papers.

After concerns were raised in 2018 regarding a potential association between dolutegravir use at conception and neural tube defects, there have been three observational studies comparing the uptake of dolutegravir between women and men in Africa. Two studies early in the rollout from found that approximately 60% of men compared to 30% of women had been transitioned to dolutegravir, while a smaller more recent study found that by September 2021, 68% of women had been transitioned to dolutegravir, compared to 80% of men.

Regarding clinical outcomes on dolutegravir, a large network meta-analysis of 68 randomised controlled trials (four of which directly compared dolutegravir 50mg daily with efavirenz among people newly initiating first-line ART) found that dolutegravir had better viral suppression, less HIV drug resistance and fewer discontinuations compared to first-line efavirenz. There are no trials assessing transition to dolutegravir among people already receiving efavirenz.

Despite the large scale dolutegravir rollout in low- and middle-income countries, there have been few assessments of the effectiveness of dolutegravir compared to other first-line regimens in non-trial settings. Among people initiating first-line ART in Brazil, 12-month viral suppression <50 copies/mL was higher among people initiated on dolutegravir versus efavirenz, but people with no 12-month viral load were excluded and loss-to-follow-up was not assessed. A retrospective cohort study among 3,108 people in four African countries found higher hazard of viraemia >1000 copies/ among people who remained on non-dolutegravir regimens compared to those transitioned to dolutegravir. However, this analysis is susceptible to multiple biases, including systematic differences in viral load schedules and the start of follow-up time between the two groups, and the use of events after baseline to determine inclusion in the time-to-event analysis.

Regarding use of dolutegravir among people with tuberculosis, rifampicin has been found to reduce dolutegravir levels. This may be overcome by doubling the dose of dolutegravir to 50mg twice daily, an approach that was found to be well tolerated in the INSPIRING trial. In non-trial settings, a cohort study among 3563 people with HIV receiving rifampicin-containing tuberculosis treatment found that those on dolutegravir-based regimens had better viral suppression compared to those who received efavirenz.

### Added value of this study

We present the largest study to date to assess dolutegravir uptake in women versus men, and to compare outcomes with non-dolutegravir first-line regimens in routine care settings. We show that younger women were less likely to be initiated or transitioned to dolutegravir early in the rollout, but this difference resolved once South African guidelines recommended dolutegravir for all people living with HIV (PLHIV), irrespective of child-bearing potential. Among both people initiating first-line ART, or already receiving first-line ART, dolutegravir use was associated with better 12-month retention and viral suppression. The association between dolutegravir and better viral suppression was even higher among people being treated for tuberculosis.

### Implications of all the available evidence

Taken together, these findings support the ongoing rollout of dolutegravir for first-line ART in low- and middle-income countries, including among people being treated for tuberculosis. Our findings also suggest that, due to initial safety concerns, women have been excluded from receiving an effective ART regimen. Therefore, particular efforts should be made to ensure that women who previously did not receive dolutegravir, are now offered dolutegravir alongside updated evidence regarding safety and effectiveness. Further evidence from routine care settings regarding adverse events, in particular weight gain, are needed.

## ABBREVIATIONS

ART: Antiretroviral therapy
aRR: Adjusted Risk Ratio
aHR: Adjusted Hazard Ratio
LMIC: Low- and Middle-Income Country
PLHIV: People living with HIV
WHO: World Health Organization
