# Supplementary material for: IMPLEMENTATION AND OUTCOMES OF DOLUTEGRAVIR-BASED FIRST-LINE ANTIRETROVIRAL THERAPY FOR PEOPLE WITH HIV IN SOUTH AFRICA: A RETROSPECTIVE COHORT STUDY
Source: Lancet HIV. Author manuscript; Available in PMC 2023 Jun 23. (PMC10288006; doi:10.1016/S2352-3018(23)00047-4)
Supplement: 3 [file NIHMS1904127-supplement-3.docx]

**Supplementary Appendix**

**Contents**

[1. Definition of first-line dolutegravir based-ART in transition cohort 1](#_Toc126219856)

[2. Emulated target trial methods 1](#_Toc126219857)

[3. Table S1 Specifications of emulated target trial 2](#_Toc126219858)

[4. Table S2 Likelihood of being initiated on dolutegravir based first line ART among women compared to men, stratified by A) age and B) time period 2](#_Toc126219859)

[5. Table S3 ‘As treated’ analysis using univariable and multivariable Poisson regression models of the association between initiation on dolutegravir and 12-month viral suppression, n = 10,097 3](#_Toc126219860)

[6. Table S4 Viral suppression among people initiated on dolutegravir versus non-dolutegravir ART, by tuberculosis status*, n = 12, 911 3](#_Toc126219861)

[7. Table S5 Rates of transition to dolutegravir by time period among people receiving non-dolutegravir based ART 3](#_Toc126219862)

[8. Table S6 Cox regression models of hazard of transition to dolutegravir among women versus men, stratified by A) age group and B) time period 4](#_Toc126219863)

[9. Table S7 Baseline characteristics of participants included in the emulated target trial, N = 92318 4](#_Toc126219864)

[10. Table S8 Poisson regression models of viral suppression among people transitioned to dolutegravir versus those not transitioned, by baseline viral load 5](#_Toc126219865)

[11. Supplementary appendix references 5](#_Toc126219866)

# Definition of first-line dolutegravir based-ART in transition cohort

Dolutegravir can be used in both first and second-line ART regimens. The standard second-line regimens in South Africa are zidovudine, lamivudine, dolutegravir, and prior to this, zidovudine, lamivudine, lopinavir/ritonavir. Therefore, in the transition cohort we classified all people changed to these regimens as being on second-line ART. The only exception was for people who were previously receiving zidovudine, lamivudine, efavirenz/nevirapine as part of their first-line regimen, and who were changed to zidovudine, lamivudine, dolutegravir; we defined this as transition to a first-line dolutegravir regimen. We also censored anyone with a viral load ≥1000 copies/mL, as they would not have been eligible for dolutegravir in a first-line regimen until they resuppressed, and may have been put on tenofovir, lamivudine, dolutegravir as part of a second-line regimen, as is advised by some clinicians.

# Emulated target trial methods

We emulated a ‘target trial’ within the transition cohort^1^ (Table S1). We fitted a Cox proportional hazards model, to estimate the log hazard of transition to dolutegravir during follow-up,^2,3^ which we then used as a time dependent propensity score. The model adjusted for gender, age, time on ART, facility, and most recent CD4 count, with interaction terms between gender and time, and gender and age. Tuberculosis, pregnancy, viral load and time in a differentiated ART program (CCMDD) were included as time dependent covariates. We included participation in a differentiated ART delivery programme^4,5^ as a time dependent co-variate, as we hypothesised that people who receive ART in the differentiated ART delivery programme may have been less likely to be transitioned to dolutegravir, as clinicians may have worried about less clinical oversight. We included viral load as a time dependent co-variate as people with low-level viraemia may have been less likely to be transitioned to dolutegravir. Individuals were censored at loss-to-follow-up, death, transfer out or if they had a viral load ≥1000 copies/mL (as they would not have been eligible at that time for transition to first-line dolutegravir). We matched people transitioned to dolutegravir with a corresponding control who had a clinic visit in the same week, but was not transitioned to dolutegravir. We used a caliper width of 0·2 times the standard deviation of the log hazard and nearest neighbour matching, and also matched directly on time since most recent viral load, and whether the participant was in a differentiated ART delivery programme. Individuals could only be matched once, and we only matched participants transitioned to dolutegravir before December 1^st^, 2020, to allow 12 months of follow-up time, plus 90 days to assess retention-in-care. This method allows definition of time zero in both the dolutegravir cohort (i.e. date of transition to dolutegravir) and their matched controls who were not transitioned to dolutegravir in the same week.^6^ Among the matched cohort, we then used multivariable Poisson regression models with robust standard errors to compare the outcomes of 12-month retention and viral suppression between the dolutegravir group and matched controls. We adjusted for gender, age, time on ART, baseline time period, most recent CD4 count, baseline viral load, and community ART delivery programme (CCMDD) use at baseline. We did not use methods that consider the data to be paired for the final analysis of outcomes.^7^

# Table S1 Specifications of emulated target trial

| **Emulated Protocol Component** | **Description** |
| --- | --- |
| Eligibility criteria | Adults >15 with HIV on first line, non-dolutegravir based antiretroviral therapy on 01 Dec 2019 at 59 clinics in eThekwini Municipality. Those with most recent viral load ≥1000 copies/mL were excluded. |
| Treatment strategies | Transitioned to dolutegravir based first line ART versus remain on non-dolutegravir based first-line ART |
| Assignment procedures | Matching 1:1 of people transitioned to dolutegravir before 01 Dec 2020, with controls selected if not transitioned to dolutegravir by the same week. Nearest neighbour matching with caliper width of 0.2 standard deviations of the log hazard, and additional matching by CCMDD use, clinic visit in same week, and time since previous viral load. |
| Follow-up period | Starts at baseline timepoint (week of matching) and ends at 15 months after baseline date (to allow assessment of 12 month outcomes, with an extra 90 days to assess retention) |
| Outcome | 1. Retention in care at 12 months 2. Viral suppression < 50 copies/mL at 12 months |
| Causal contrasts of interest | Intention-to-treat effect, with sensitivity analysis assessing per-protocol effect (excluding people who changed from or to dolutegravir-based ART during follow-up) |
| Analysis plan | Multivariable Poisson regression model with robust standard errors to account for clustering by clinic. |

CCMDD = Centralised Chronic Medicines Dispensing and Distribution programme

# Table S2 Likelihood of being initiated on dolutegravir based first line ART among women compared to men, stratified by A) age and B) time period

|  | **Men** | **Women, non-pregnant** | | **Women, pregnant** | |
| --- | --- | --- | --- | --- | --- |
|  | **(n/N)** | **(n/N)** | **RR (95% CI)** | **(n/N)** | **RR (95% CI)** |
| **A) Age (years)*** |  |  |  |  |  |
| ≥55 | 431/555 (77·7%) | 462/615 (75·1%) | 0·97 (0·90-1·03) | - | - |
| 45-54 | 1620/2005 (80·8%) | 1321/1789 (73·8%) | 0·91 (0·87-0·96) | - | - |
| 35-44 | 5091/6185 (82·3%) | 3308/5040 (65·6%) | 0·80 (0·76-0·83) | - | - |
| 25-34 | 5642/6805 (82·9%) | 6747/10832 (59·0%) | 0·75 (0·71-0·80) | - | - |
| 15-24 | 935/1117 (83·7%) | 3468/5680 (61·1%) | 0·73 (0·69-0·77) | - | - |
| **B) Time period** |  |  |  |  |  |
| Dec 19 - Feb 20 | 926/2660 (34·8%) | 390/3808 (10·2%) | 0·29 (0·23-0·38) | 61/706 (8·6%) | 0·25 (0·12-0·53) |
| Mar 20 - May 20 | 1647/2104 (78·3%) | 906/2875 (31·5%) | 0·40 (0·33-0·49) | 76/603 (12·6%) | 0·16 (0·08-0·32) |
| Jun 20 - Aug 20 | 1529/1714 (89·2%) | 1553/2653 (58·5%) | 0·66 (0·58-0·74) | 128/573 (22·5%) | 0·25 (0·16-0·40) |
| Sep 20 - Nov 20 | 1690/1859 (90·9%) | 1753/2657 (66·0%) | 0·73 (0·66-0·80) | 209/609 (34·3%) | 0·38 (0·27-0·52) |
| Dec 20 - Feb 21 | 1590/1725 (92·2%) | 1853/2413 (76·8%) | 0·83 (0·78-0·90) | 284/525 (54·1%) | 0·59 (0·49-0·70) |
| Mar 21 - May 21 | 2020/2105 (96·0%) | 2716/3076 (88·3%) | 0·92 (0·88-0·96) | 330/487 (67·8%) | 0·71 (0·63-0·79) |
| Jun 21 - Aug 21 | 1427/1485 (96·1%) | 2088/2273 (91·9%) | 0·96 (0·92-0·99) | 380/458 (83·0%) | 0·86 (0·80-0·94) |
| Sep 21 - Nov 21 | 1441/1509 (95·5%) | 2040/2125 (96·0%) | 1·00 (0·98-1·03) | 402/429 (93·7%) | 0·98 (0·94-1·02) |
| Dec 21 - Feb 22 | 1449/1506 (96·2%) | 2007/2076 (96·7%) | 1·00 (0·99-1·02) | 369/379 (97·4%) | 1·01 (0·99-1·04) |

*Pregnant women excluded as there were too few pregnant women in older age groups

Wald test for age-gender interaction, p < 0.001, and time period-gender interaction, p <0.001

# Table S3 ‘As treated’ analysis using univariable and multivariable Poisson regression models of the association between initiation on dolutegravir and 12-month viral suppression, n = 10,097

| **Variable** | **Levels** | **Viral suppression** | **RR** | **aRR*** |
| --- | --- | --- | --- | --- |
| ART regimen | Non-DTG regimen | 3187 (78·7) | 1 | 1 |
|  | DTG regimen | 5029 (83·2) | 1·06 (1·03-1·09) | 1·09 (1·05-1·12^†^ |
| Gender | Male | 2998 (78·9) | 1 | 1 |
|  | Female, not pregnant | 4439 (83·0) | 1·05 (1·04-1·07) | 1·07 (1·05-1·09) |
|  | Female, pregnant | 779 (82·4) | 1·05 (1·01-1·08) | 1·08 (1·04-1·13) |
| Age (years) | 55+ | 230 (82·4) | 1 | 1 |
|  | 45-54 | 799 (82·7) | 1·00 (0·94-1·07) | 1·01 (0·95-1·08) |
|  | 35-44 | 2238 (80·8) | 0·98 (0·93-1·04) | 1·00 (0·94-1·06) |
|  | 25-34 | 3654 (81·5) | 0·99 (0·93-1·05) | 0·99 (0·93-1·05) |
|  | 15-24 | 1295 (81·0) | 0·98 (0·93-1·04) | 0·97 (0·91-1·03) |
| Initiation time period | Dec 19 - Feb 20 | 2167 (78·1) | 1 | 1 |
|  | Mar 20 - May 20 | 2172 (82·9) | 1·06 (1·03-1·09) | 1·04 (1·01-1·07) |
|  | Jun 20 - Aug 20 | 1988 (84·8) | 1·09 (1·05-1·12) | 1·04 (1·01-1·08) |
|  | Sep 20 - Nov 20 | 1889 (80·0) | 1·02 (0·99-1·06) | 0·98 (0·95-1·02) |
| TB at ART initiation | No TB | 7770 (81·7) | 1 | 1 |
|  | Known TB | 446 (76·1) | 0·93 (0·89-0·98) | 1·00 (0·95-1·04) |
| Initiation CD4 count (cells/µL) | <200 | 1394 (72·7) | 1 | 1 |
|  | 200-349 | 1542 (80·0) | 1·10 (1·06-1·14) | 1·10 (1·06-1·14) |
|  | 350-499 | 1320 (83·2) | 1·14 (1·10-1·19) | 1·14 (1·10-1·19) |
|  | >=500 | 2204 (87·9) | 1·21 (1·17-1·24) | 1·20 (1·17-1·24) |
|  | Missing | 1756 (81·4) | 1·12 (1·08-1·16) | 1·11 (1·08-1·15) |

*The primary exposure effect (dolutegravir use) is adjusted for all other variables in the model as potential confounders. Unlike the primary exposure effect, the presented adjusted risk ratios for potential confounding variables should not be interpreted as the effect of the confounding variable on the outcome. ^†^Estimated risk difference 6.8%, 95% CI 4.3-9.4.

# Table S4 Viral suppression among people initiated on dolutegravir versus non-dolutegravir ART, by tuberculosis status*, n = 12, 911

|  | **Proportion with viral suppression <50 copies/mL** | |  |
| --- | --- | --- | --- |
| **TB status at ART initiation** | **Non-dolutegravir regimen (n/N)** | **Dolutegravir regimen (n/N)** | **aRR (95% CI)** |
| No TB | 5000/6083 (82·2%) | 5032/6054 (83·1%) | 1·03 (1·01-1·06) |
| Known TB | 327/458 (71·4%) | 257/316 (81·3%) | 1·14 (1·07-1·22) |

Wald test for interaction, p = 0.006

*Multivariable Poisson regression model adjusted for age, gender, time, initiation time period and initiation CD4 count

# Table S5 Rates of transition to dolutegravir by time period among people receiving non-dolutegravir based ART

| Time period | Events | Time (person-years) | Event rate per 1000 person-years (95% CI) |
| --- | --- | --- | --- |
| Dec 19 - Feb 20 | 2361 | 44300 | 53.3 (51.2-55.5) |
| Mar 20 - May 20 | 19791 | 40254 | 491.6 (484.8-498.5) |
| Jun 20 - Aug 20 | 35094 | 31371 | 1118.6 (1107.0-1130.4) |
| Sep 20 - Nov 20 | 17977 | 22870 | 786.0 (774.6-797.6) |
| Dec 20 - Feb 21 | 6746 | 19534 | 345.3 (337.1-353.7) |
| Mar 21 - May 21 | 11820 | 16507 | 716.0 (703.2-729.0) |
| Jun 21 - Aug 21 | 10936 | 13150 | 831.6 (816.1-847.3) |
| Sep 21 - Nov 21 | 10009 | 10080 | 993.0 (973.6-1012.6) |
| Dec 21 - Feb 22 | 6440 | 7960 | 809.0 (789.4-829.0) |

# Table S6 Cox regression models of hazard of transition to dolutegravir among women versus men, stratified by A) age group and B) time period

|  | **Proportion transitioned to dolutegravir** | |  |
| --- | --- | --- | --- |
|  | **Women (n/N)** | **Men (n/N)** | **aHR (95% CI)** |
| A) Age group (years)* | | | |
| ≥55 | 6423/8886 (72·3%) | 3652/5124 (71·3%) | 0·93 (0·90-0·97) |
| 45-54 | 15371/20707 (74·2%) | 10349/13516 (76·6%) | 0·76 (0·74-0·78) |
| 35-44 | 28460/43198 (65·9%) | 18063/23920 (75·5%) | 0·54 (0·53-0·55) |
| 25-34 | 24970/43351 (57·6%) | 8874/12593 (70·5%) | 0·48 (0·47-0·49) |
| 15-24 | 4007/8026 (49·9%) | 1041/1635 (63·7%) | 0·50 (0·46-0·53) |
| B) Time period**^†^** | | | |
| Dec 19 - Feb 20 | 1056/124168 | 1305/56788 | 0·37 (0·34-0·40) |
| Mar 20 - May 20 | 9096/119157 | 10696/53646 | 0·35 (0·34-0·36) |
| Jun 20 - Aug 20 | 20813/105885 | 14286/40886 | 0·50 (0·49-0·51) |
| Sep 20 - Nov 20 | 11929/81235 | 6049/24966 | 0·57 (0·55-0·58) |
| Dec 20 - Feb 21 | 4697/65529 | 2049/17469 | 0·59 (0·56-0·62) |
| Mar 21 - May 21 | 9100/58794 | 2727/14666 | 0·81 (0·78-0·84) |
| Jun 21 - Aug 21 | 8778/47947 | 2172/11380 | 0·94 (0·90-0·99) |
| Sep 21 - Nov 21 | 8259/37886 | 1755/8758 | 1·09 (1·04-1·15) |
| Dec 21 - Feb 22 | 5503/28568 | 940/6661 | 1·39 (1·30-1·49) |

*Likelihood ratio test for interaction p < 0·001, **^†^**Likelihood ratio test for interaction p < 0·001

# Table S7 Baseline characteristics of participants included in the emulated target trial, N = 92318

| **Variable** | **Levels** | **No DTG** | **DTG** | **Total** |
| --- | --- | --- | --- | --- |
| Gender | Male | 17860 (38.7) | 17626 (38.2) | 35486 (38.4) |
|  | Female | 28299 (61.3) | 28533 (61.8) | 56832 (61.6) |
| Baseline age (cont.), years | Median (IQR) | 39.0 (33.0 to 46.0) | 40.0 (34.0 to 47.0) | 39.0 (33.0 to 47.0) |
| Baseline age (cat.), years | 55+ | 4250 (9.2) | 4657 (10.1) | 8907 (9.6) |
|  | 45-54 | 10378 (22.5) | 10795 (23.4) | 21173 (22.9) |
|  | 35-44 | 17518 (38.0) | 17656 (38.3) | 35174 (38.1) |
|  | 25-34 | 12093 (26.2) | 11470 (24.8) | 23563 (25.5) |
|  | 15-24 | 1920 (4.2) | 1581 (3.4) | 3501 (3.8) |
| Baseline time on ART, years | Median (IQR) | 4.0 (1.9 to 6.6) | 4.2 (2.3 to 6.7) | 4.1 (2.1 to 6.7) |
| Most recent CD4 count (cont.), cells/µL | Median (IQR) | 443.0 (290.0 to 627.0) | 445.0 (292.0 to 627.0) | 444.0 (291.0 to 627.0) |
| Most recent CD4 count (cat.), cells/µL | >=500 | 17397 (37.7) | 17639 (38.2) | 35036 (38.0) |
|  | 350-499 | 10505 (22.8) | 10458 (22.7) | 20963 (22.7) |
|  | 200-349 | 8951 (19.4) | 9125 (19.8) | 18076 (19.6) |
|  | <200 | 5704 (12.4) | 5512 (11.9) | 11216 (12.1) |
|  | Missing | 3602 (7.8) | 3425 (7.4) | 7027 (7.6) |
| Baseline time period | Dec 19 - Feb 20 | 1439 (3.1) | 1439 (3.1) | 2878 (3.1) |
|  | Mar 20 - May 20 | 13607 (29.5) | 13607 (29.5) | 27214 (29.5) |
|  | Jun 20 - Aug 20 | 21503 (46.6) | 21503 (46.6) | 43006 (46.6) |
|  | Sep 20 - Nov 20 | 9610 (20.8) | 9610 (20.8) | 19220 (20.8) |
| TB at baseline? | No | 46063 (99.8) | 46085 (99.8) | 92148 (99.8) |
|  | Yes | 96 (0.2) | 74 (0.2) | 170 (0.2) |
| Pregnant at baseline? | No | 46059 (99.8) | 46042 (99.7) | 92101 (99.8) |
|  | Yes | 100 (0.2) | 117 (0.3) | 217 (0.2) |
| Most recent viral load at baseline (copies/mL) | <50 | 42614 (92.3) | 42782 (92.7) | 85396 (92.5) |
|  | 50-199 | 1665 (3.6) | 1651 (3.6) | 3316 (3.6) |
|  | 200-399 | 505 (1.1) | 423 (0.9) | 928 (1.0) |
|  | 400-999 | 464 (1.0) | 392 (0.8) | 856 (0.9) |
|  | Missing | 911 (2.0) | 911 (2.0) | 1822 (2.0) |
| In CCMDD at baseline | No | 32224 (69.8) | 32224 (69.8) | 64448 (69.8) |
|  | Yes | 13935 (30.2) | 13935 (30.2) | 27870 (30.2) |

# Table S8 Poisson regression models of viral suppression among people transitioned to dolutegravir versus those not transitioned, by baseline viral load

|  | **Proportion with viral suppression** | |  |
| --- | --- | --- | --- |
| **Baseline viral load (copies/ml)** | **Dolutegravir (n/N)** | **Non-dolutegravir (n/N)** | **aRR (95% CI)** |
| Intention-to-treat analysis* |  |  |  |
| <50 | 31414/34477 (91·1%) | 29843/32921 (90·7%) | 1·01 (1·00-1·01) |
| 50-199 | 1082/1301 (83·2%) | 997/1239 (80.5%) | 1·03 (1·00-1·07) |
| 200-399 | 265/329 (80·5%) | 275/394 (69·8%) | 1·15 (1·05-1·26) |
| 400-999 | 255/309 (82·5%) | 221/335 (66·0%) | 1·25 (1·13-1·38) |
| Missing | 407/504 (80·8%) | 312/410 (76·1%) | 1·06 (0·97-1·16) |
| As-treated analysis |  |  |  |
| <50 | 31351/34408 (91·1%) | 13275/14766 (89·9%) | 1·02 (1·01-1·02) |
| 50-199 | 1080/1299 (83·1%) | 417/543 (76.8%) | 1·08 (1·02-1·15) |
| 200-399 | 263/327 (80·4%) | 108/187 (57·8%) | 1·39 (1·22-1·58) |
| 400-999 | 254/308 (82·5%) | 81/162 (50·0%) | 1·65 (1·40-1·95) |
| Missing | 403/500 (80·6%) | 134/196 (68·4%) | 1·18 (1·04-1·32) |

*Wald test for interaction p<0.001, **^†^**Wald test for interaction p<0.001

# Supplementary appendix references

1. Hernán MA, Robins JM. Using Big Data to Emulate a Target Trial When a Randomized Trial Is Not Available. *American Journal of Epidemiology* 2016; **183**(8): 758-64.

2. Lu B. Propensity score matching with time-dependent covariates. *Biometrics* 2005; **61**(3): 721-8.

3. Thomas LE, Yang S, Wojdyla D, Schaubel DE. Matching with time-dependent treatments: A review and look forward. *Stat Med* 2020; **39**(17): 2350-70.

4. Dorward J, Msimango L, Gibbs A, et al. Understanding how community antiretroviral delivery influences engagement in HIV care: a qualitative assessment of the Centralised Chronic Medication Dispensing and Distribution programme in South Africa. *BMJ Open* 2020; **10**(5): e035412.

5. Liu L, Christie S, Munsamy M, et al. Expansion of a national differentiated service delivery model to support people living with HIV and other chronic conditions in South Africa: a descriptive analysis. *BMC Health Services Research* 2021; **21**(1): 463.

6. Garcia-Albeniz X, Hsu J, Hernan MA. The value of explicitly emulating a target trial when using real world evidence: an application to colorectal cancer screening. *Eur J Epidemiol* 2017; **32**(6): 495-500.

7. Stuart EA. Developing practical recommendations for the use of propensity scores: discussion of 'A critical appraisal of propensity score matching in the medical literature between 1996 and 2003' by Peter Austin, Statistics in Medicine. *Stat Med* 2008; **27**(12): 2062-5; discussion 6-9.
